# Smurf2 suppresses the metastasis of hepatocellular carcinoma via ubiquitin degradation of Smad2

**DOI:** 10.1515/med-2022-0437

**Published:** 2022-02-24

**Authors:** Dongqiang Song, Shuyu Li, Liuxin Ning, Shuncai Zhang, Yu Cai

**Affiliations:** Liver Cancer Institute, Zhongshan Hospital of Fudan University, Xuhui District, Shanghai, P. R. China; Department of Gastroenterology and Hepatology, Zhongshan Hospital of Fudan University, Xuhui District, Shanghai, P. R. China

**Keywords:** Smurf2, hepatocellular carcinoma, Smad2, ubiquitination, metastasis

## Abstract

**Purpose:**

Smurf2, one of C2-WW-HECT domain E3 ubiquitin ligases, is closely related to the development and progression in different cancer types, including hepatocellular carcinoma (HCC). This study aims to illustrate the expression and molecular mechanism of Smurf2 in regulating the progression of HCC.

**Methods:**

The expression of Smurf2 in human HCC and adjacent non-tumor liver specimens was detected using tissue microarray studies from 220 HCC patients who underwent curative resection. The relationships of Smurf2 and HCC progression and survival were analyzed using the chi-square test, Kaplan–Meier analysis, and Cox proportional hazards model. For Smurf2 was low expression in HCC cell lines, Smurf2 overexpression cell lines were established. The effect of Smurf2 on cell proliferation and migration was detected by Cell Counting Kit-8 and colony formation assay, and the epithelial–mesenchymal transition (EMT) markers and its transcription factors were tested by immunoblotting. The interaction and ubiquitination of Smad2 by Smurf2 were detected by co-immunoprecipitation and immunoprecipitation assay. Finally, the effect of Smurf2 on HCC was verified using the mouse lung metastasis model.

**Results:**

Smurf2 was downregulated in HCC tissues compared to that of corresponding non-tumor liver specimens. The low expression of Smurf2 in HCC was significantly associated with macrovascular or microvascular tumor thrombus and the impairment of overall survival and disease-free survival. *In vitro* and *in vivo* analysis showed that Smurf2 overexpression decreased the EMT potential of HCC cells by promoting the ubiquitination of Smad2 via the proteasome-dependent degradation pathway.

**Conclusion:**

The expression of Smurf2 was downregulated in HCC specimens and affected the survival of patients. Smurf2 inhibited the EMT of HCC by enhancing Smad2 ubiquitin-dependent proteasome degradation.

## 1 Introduction

Hepatocellular carcinoma (HCC) contributes to the third most common cause of cancer-related mortality in the world [1]. The 5-year survival rate is less than 20%, with little change in the past few years. Although significant progress has been made in the diagnosis and treatment of HCC in recent decades, the long-term clinical prognosis and mortality are still unsatisfactory [2]. Due to the high recurrence rate and increased drug resistance, the prognosis of most patients is still not ideal [3]. Therefore, it is imperative to study new therapeutic targets. Understanding the molecular biology of HCC is crucial for selecting the most appropriate treatment and improving the clinical outcome of HCC management.

The ubiquitin–proteasome pathway is an important protein degradation pathway with high selectivity in eukaryotes. It can efficiently and selectively degrade intracellular proteins, participate in cell signal transduction, and play an important role in cell metabolism, proliferation, and differentiation [4]. When some target proteins of the ubiquitin–proteasome pathways, such as cell cycle regulatory proteins, oncogenes, or tumor suppressor genes, are degraded abnormally, cell proliferation is accelerated, apoptosis is blocked, and finally, tumorigenesis occurs [5]. The ubiquitin–proteasome pathway is mediated by three main enzymes: ubiquitin-activating enzyme (E1), ubiquitin-conjugating enzyme (E2), and ubiquitin-protein ligase (E3). E3 ligase is the major component of the ubiquitin cascade [6].

Smurf2 (Smad2 ubiquitination regulation factor 2) is one of C2-WW-HECT domain E3 ubiquitin ligases, which are thought to mediate E3 and the interaction of the substrates through association with PPxY or LPxY motifs containing proline in the binding partner [7]. Smurf2 is closely related to the development and progression of tumors. For example, the expression of Smurf2 increased in esophageal squamous cell carcinoma and pancreatic carcinoma, which was related to tumor invasion and lymph node metastasis [8]. Overexpression of Smurf2 can promote invasion and metastasis in breast cancer [9]. Some studies have found that the substrate proteins of Smurf2 include Smad2/3, Smad7, and transforming growth factor-β (TGF-β) receptor and nuclear repressor ski family factor SnoN [10]. These substrate proteins are involved in the regulation of the TGF-β-Smad signaling pathway. Smurf2 regulates TGF-β through the following two pathways: (A) Smad2, as a regulator of Smurf2 pathway, promotes the combination of Smurf2 and SnoN, which leads to the ubiquitination and degradation of SnoN; (B) Smad7, as a regulator of Smurf2 pathway, binds to Smurf2 and then transfers to the cytoplasm to activate TGF-β receptor degradation. However, it is unclear how Smurf2 affects liver disease. Our team used a gene chip to screen the genes in the process of rat liver fibrosis model and found that Smurf2 was abnormally expressed in the process of liver fibrosis, which proved that Smurf2 regulates TGF-β signaling pathways to affect the occurrence and development of liver fibrosis [11]. However, the relationship between Smurf2 and HCC remains unclear.

In the present study, we detected the expression of Smurf2 in human HCC and adjacent non-tumor liver specimens, discussed the relationship between Smurf2 and clinicopathological features of HCC patients, and investigated the tumor-inhibiting effects of Smurf2 *in vitro* and *in vivo*, as well as its underlying mechanism involved with ubiquitination of Smad2.

## 2 Materials and methods

### 2.1 Patients and tissue specimens

As clinical specimens for tissue microarray (TMA) studies, the 220 pairs of HCC tissues and adjacent non-tumor tissues were derived from HCC patients who underwent curative resection in Zhongshan Hospital of Fudan University. The patients with HCC include 186 male and 34 female. Complete follow-up data were obtained for all patients, and the diagnosis of HCC was confirmed by pathological examination. Patient samples were collected with the approval of the Research Ethics Committee of Zhongshan Hospital. From all participants was obtained informed consent.

Immunohistochemistry (IHC) analysis was performed to confirm Smurf2 expression. The expression level was independently evaluated by two authorized pathologists according to the intensity and proportion of positive cells. The staining intensity was evaluated under a light microscope on a 4-point scale: 0, no staining; 1, weak staining; 2, intermediate staining; and 3, strong staining, and the percentage of positively stained cells were divided as 0, 0%; 1, 1–25%; 2, 26–50%; 3, 51–75%; and 4, 76–100%. The final expression score was calculated by multiplying the intensity score with the percentage score of positive cells. An overall score of 12 was acquired and graded as score 0, negative; score 1–4, weak; score 5–8, moderate; and score 9–12, strong.


**Ethical approval and consent to participate:** Approval and consent obtained for the use of human tissue and all animal procedures were obtained from the Research Ethics Committee and the Animal Care Committee of Zhongshan Hospital of Fudan University, The ethics approval number: B2021-659 R.

### 2.2 Cell lines and animals

The human HCC cell lines Hep3B and Huh7 were obtained from the Cell Bank of the Chinese Academy of Sciences (Shanghai, China). HCCLM3 and MHCC97H were obtained from the Liver Cancer Institute, Zhongshan Hospital of Fudan University. All cells were cultured in Dulbecco’s Modified Eagle Medium (HyClone, Logan, UT, USA) supplemented with 10% fetal bovine serum (FBS, HyClone) a 37°C humidified atmosphere with 5% CO_2_.

Male athymic BALB/c nude mice were purchased from Slack Laboratory Animal Co., Ltd (Shanghai, China). The animal maintenance and experimental procedures were approved by the Institutional Animal Care and Use Committee of Fudan University.

### 2.3 Cell transfection

Cells overexpressing Smurf2 and the matched control cells were established using lentivirus carrying the PCDH-EF1a-Puro plasmid (Genepharma, Shanghai, China). Puromycin (2 µg/mL) was used to select the cells stably transfected with Smurf2. The efficiency of gene overexpression was evaluated by western blotting.

### 2.4 *In vivo* models

Twelve BABL/c nude mice aged 4 weeks, purchased from Slack Laboratory Animal Co., Ltd (Shanghai, China), were kept in the specific pathogen-free condition. For the mouse lung metastasis model, mice were randomly divided into two groups: overexpressing Smurf2 and Smurf2 NC group, respectively. Around 1  ×  10^6^ Huh7 cells overexpressing Smurf2 and the control cells were injected into nude mice (*n*  =  6 per group) through the tail vein. After 6 weeks, the mice were scanned by 18F-FDG positron emission tomography (PET) (MedicLab PET/MR, Madic Technology Co. Ltd, Shandong, China) to observe a general picture of the tumor metastasis. Then the mice were sacrificed, and the lungs were excised, imaged, and fixed in formalin, embedded in paraffin. Afterward, the lung tissues were embedded in paraffin to be cut. Tumor metastases were confirmed by hematoxylin and eosin (H&E) staining for later metastatic nodules calculation. All animal experiments were approved by the Institutional Animal Care and Use Committee of Zhongshan hospital, Fudan University.

### 2.5 Cell proliferation assay

Hep3B or huh7 cells that stably expressing Smurf2 were seeded into 96-well plates with 5 × 10^3^ cells/well. After 24, 48, and 72 h, the cells were incubated with Cell Counting Kit-8 (CCK8) solution (Beyotime, Jiangsu, China) for 2 h at 37°C. Then, the product was quantified spectrophotometrically at a wavelength of 450 nm using a microplate reader (Bio-Rad, California, USA). Experiments were conducted with six replicates and repeated three times.

### 2.6 Colony formation assay

One thousand Hep3B or huh7 cells that stably expressing Smurf2 were seeded into 6-well plates. Two weeks later, plates were fixed with 4% paraformaldehyde (Merck, Darmstadt, Germany) at room temperature for 15 min, and stained with 0.1% crystal violet (Beyotime, Jiangsu, China). Images were obtained by a camera (Sony, Tokyo, Japan), and the number of colonies was counted and calculated.

### 2.7 Transwell migration assay

The transwell chambers were prepared as 8 µm pores (3422, Corning, USA). Hep3B or huh7 cells stably expressing Smurf2 were seeded into the upper chambers with 5 × 10^4^ cells/well, while the lower chambers were filled with 600 µL DMEM medium containing 20% serum. Afterward, the cells on the top of the membrane are removed, and the cells which migrated to the lower chambers were fixed with 4% paraformaldehyde and stained with crystal violet before counted with an inverted microscope (BX51, Olympus, Japan) 24 h later.

### 2.8 Co-immunoprecipitation (Co-IP) and immunoprecipitation

A Co-IP assay was done as previously described [12]. Briefly, cells were lysed in 500 µL Co-IP buffer (50 mM Tris-HCl, 150 mM NaCl, 10 mM EDTA, and 1% NP-40, pH 7.8) supplemented with a protease inhibitor cocktail (P1048, Beyotime, China). Subsequently, the cell lysates were centrifuged and incubated with anti-Smad2 antibody (1:100 dilution; AF1300; Beyotime, China) and Protein G agarose beads (L-1006, Biolinkedin Biotech Co., Ltd, Shanghai, China) overnight at 4°C. For immunoprecipitation, cells were lysed in 500 µL immunoprecipitation buffer (50 mM Tris-HCl, 150 mM NaCl, 5 mM EDTA, 0.1% SDS, and 1% NP-40, pH 7.8) supplemented with a protease inhibitor cocktail. Subsequently, the cell lysates were centrifuged and incubated with anti-Smad2 antibody and Protein G agarose beads overnight at 4°C. The immunoprecipitates were enriched and denatured at 100°C for 10 min in 2X sodium dodecyl sulfate–polyacrylamide gel electrophoresis (SDS-PAGE) loading buffer (50 µL). The inputs, immunoprecipitants, and other cell lysates were performed by western blotting analysis.

### 2.9 Western blotting analysis

Western blotting analysis was done as previously described [13,14]. Total protein extraction was disintegrated using RIPA lysis buffer (P0013; Biotime, China) and quantified using the BCA protein assay kit (P0010; Beyotime, China). Then subjected to SDS-PAGE and transferred to polyvinylidene fluoride membrane (Bio-Rad, USA), which was incubated with the primary antibodies against Smurf2 (1:1,000, 12,024 s; Cell Signaling, USA), Smad2 (1:1,000, AF1300; Beyotime, China), GAPDH (1:1,000, 2118; Cell Signaling, USA), Ubiquitin (1:1,000, 3933 s; Cell Signaling, USA), E-cadherin (1:1,000, 20874-1-AP; Proteintech, China), ZO-1 (1:1,000, 5406; Cell Signaling, USA), Claudin1 (1:1,000, 5406; Cell Signaling, USA), N-cadherin (1:1,000, 22018-1-AP; Proteintech, China), Vimentin (1:2000, 10366-1-AP; Proteintech, China), twist (1:1,000, 25465-1-AP; Proteintech, China), slug (1:1,000, 12129-1-AP; Proteintech, China), snail (1:1,000, 13099-1-AP; Proteintech, China), and β-actin (1:1,000, D110001; Sangon Biotech, China). The secondary antibodies were labeled with horseradish peroxidase (7074 s, Cell Signaling, USA), and the signals were visualized using Tanon 5200 Imaging System (Tanon, China).

### 2.10 Statistical analysis

All experiments were performed in triplicate. Statistical analyses were performed with SPSS 23.0 software (SPSS Inc., Chicago, IL, USA). Means were compared between two groups using unpaired, two-tailed Student’s *t*-test, and among multiple groups using one-way analysis of variance. Categorical data were analyzed using *χ*2 or Fisher’s exact tests. The survival curve analysis was assessed by the Kaplan–Meier method and differences were assessed by the log-rank test. Univariate and multivariate analyses were performed using the Cox proportional hazards regression model. *P < 0.05 was considered to be significantly difference; **P < 0.01 was considered to be very significantly difference.

## 3 Results

### 3.1 Decreased Smurf2 expression in HCC tissues and the low expression of Smurf2 associated with poor prognosis

**Figure 1:**
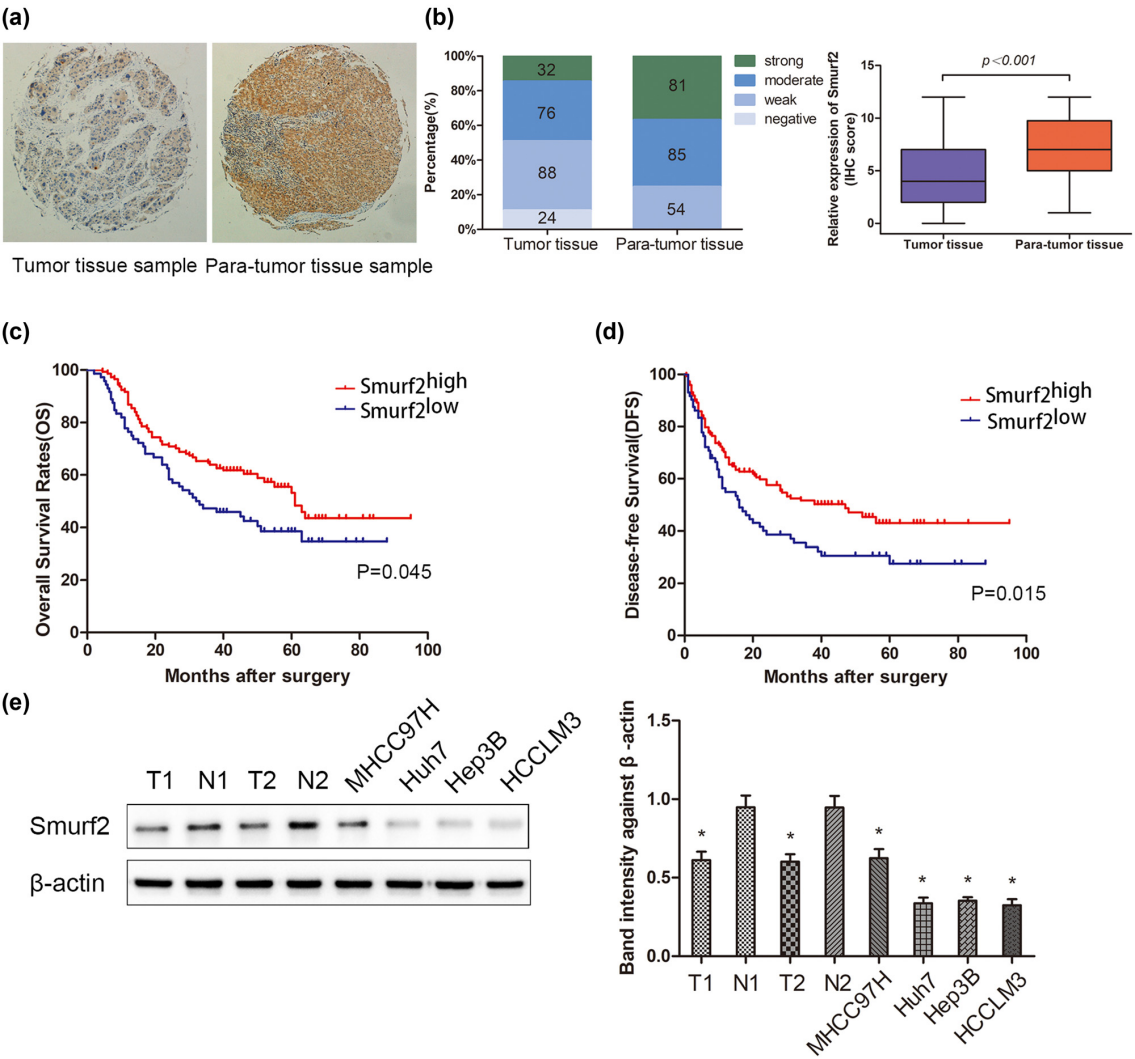
Smurf2 expression in HCC tissues and adjacent non-tumor tissues. (a) Representative immunohistochemical staining of smurf2 in tumor tissue and para-tumor tissue. Magnification, ×200. (b) IHC score in HCC tissue and para-tumor tissue (*n* = 220; *P* < 0.001). (c and d) OS curve and DFS curve for HCC patients with high vs low expression of Smurf2 IHC score generated with Kaplan–Meier methods. (e) The immunoblotting of Smurf2 protein levels in HCC tissues (T), adjacent non-tumor liver tissues (N), and human HCC cell lines. *P < 0.05.

TMA was used to evaluate the correlation between Smurf2 and prognosis in 220 HCC patients who underwent curative resection. As shown in Figure 1a, the human HCC tissues presented a low expression of Smurf2 as compared to that of adjacent non-tumor liver tissues. The comparison of the relative expression of Smurf2 expression between tumor tissue and adjacent tissue indicated that Smurf2 was downregulated in HCC tumor tissue compared with adjacent tissue (Figure 1b).

The clinical–pathological data of 220 patients were collected for the chi-square test, to explore the correlation between Smurf2 expression and clinical pathological indexes. According to the Smurf2 expression score of IHC staining in HCC tissues, patients were divided into the Smurf2 high expression group (greater than or equal to 4 points) and low expression group (less than 4 points). As shown in Table 1, the expression of Smurf2 was significantly correlated with macrovascular or microvascular tumor thrombus (*P* = 0.045). There was no significant correlation between the expression of Smurf2 and gender, age, hepatitis B surface antigen (HBsAg), α-fetoprotein (AFP), liver cirrhosis, tumor number, tumor size, tumor encapsulation, tumor differentiation or tumor-nodes-metastasis (TNM) stage, and Barcelona Clinic Liver Cancer (BCLC) stage.

**Table 1:** Clinical characteristics of 220 HCC patients

Clinicopathological indexes		220 HCC patients		*P*-values
		Smurf2 low (72)	Smurf2 high (148)	
Sex	Female	15	19	0.124
	Male	57	129	
Age (years)	≤50	41	71	0.212
	＞50	31	77	
HBsAg	Negative	4	12	0.157
	Positive	59	128	
AFP	≤20	26	50	0.733
	＞20	46	98	
Liver cirrhosis	No	8	19	0.714
	Yes	64	129	
Tumor number	Single	53	116	0.432
	Multiple	19	32	
Tumor size (cm)	≤5	31	78	0.179
	＞5	41	70	
Tumor encapsulation	Complete	35	74	0.847
	None	37	74	
Macrovascular or microvascular tumor thrombus	No	35	92	0.045
	Yes	37	56	
Tumor differentiation	I–II	35	90	0.087
	III–IV	37	58	
TNM stage	I	44	104	0.174
	II–III	28	44	
BCLC stage	0–A	34	90	0.057
	B–C	38	58	

*P*-value <0.05 was considered, statistically significant. *P*-values were calculated using the Pearson chi-square test.

Next, we investigated the relationship between Smurf2 expression and prognosis in the HCC patients, the followed-up period ranged from 1.3 to 95 months. The overall survival (OS) and disease-free survival (DFS) were compared between the patients with low Smurf2 expression (*n* = 72) and those with high Smurf2 expression (*n* = 148) by Kaplan–Meier survival analysis. The result showed that low Smurf2 expression predicted a worse OS (*p* = 0.045) and DFS (*p* = 0.015) in the HCC patients (Figure 1c and d). Cox regression model for OS showed that macrovascular or microvascular tumor thrombus and tumor differentiation were significantly associated with an increased risk of cancer-related death, while Smurf2 expression was significantly associated with decreased risk of cancer-related death (Table 2). Furthermore, tumor number and macrovascular or microvascular tumor thrombus were significantly associated with poor DFS for the HCC patients (Table 3). These data suggested that the loss of Smurf2 expression may be an important role in the progression of human HCC.

**Table 2:** Univariate and multivariate analyses of clinicopathological factors for OS in HCC patients

Characteristics	Univariate analysis			Multivariate analysis		
	HR	95% CI	*P*	HR	95% CI	*P*
Sex	0.801	0.465–1.38	0.423			
Age (years)	0.949	0.655–1.375	0.783			
HBsAg	1.058	0.669–1.674	0.81			
AFP (ng/mL, ≤20 vs ＞20)	2.197	1.422–3.397	0.001			0.175
Liver cirrhosis (no vs yes)	1.98	1.001–3.917	0.045			0.187
Tumor number (single vs multiple)	1.998	1.337–2.985	0.001			0.143
Tumor size (cm, ≤5 vs ＞5)	2.352	1.598–3.462	0.001			0.554
Tumor encapsulation (complete vs none)	1.552	1.066–2.26	0.021			0.81
Macrovascular or microvascular tumor thrombus (no vs yes)	3.998	2.71–5.9	0.001	4.056	2.735–6.017	0.001
Tumor differentiation (I–II vs III–IV)	1.916	1.321–2.779	0.001	1.707	1.174–2.481	0.005
Smurf2	0.529	0.315–0.888	0.016	0.42	0.249–0.708	0.001

HR, hazard ratio; CI, confidential interval; *P*-value <0.05 was considered statistically significant. Cox proportional hazards regression model.

**Table 3:** Univariate and multivariate analyses of clinicopathological factors for DFS in HCC patients

Factors	Univariate analysis			Multivariate analysis		
	HR	95% CI	*P*	HR	95% CI	*P*
Sex	0.6	0.344–1.047	0.069			
Age (years)	0.873	0.615–1.241	0.45			
HBsAg	1.33	0.866–2.042	0.194			
AFP (ng/mL, ≤20 vs ＞20)	1.832	1.238–2.71	0.002			0.116
Liver cirrhosis (no vs yes)	1.45	0.816–2.576	0.202			
Tumor number (single vs multiple)	2.191	1.498–3.205	0.001	2.034	1.388–2.982	0.001
Tumor size (cm, ≤5 vs ＞5)	2.167	1.509–3.114	0.001			0.509
Tumor encapsulation (complete vs none)	1.837	1.284–2.629	0.001			0.17
Macrovascular or microvascular tumor thrombus (no vs yes)	3.918	2.718–5.648	0.001	3.812	2.64–5.504	0.001
Tumor differentiation (I–II vs III–IV)	1.418	0.997–2.019	0.051			0.368
Smurf2	0.645	0.45–0.925	0.017			0.196

*P*-value <0.05 was considered statistically significant. Cox proportional hazards regression model.

### 3.2 Low expression of Smurf2 in human HCC cell lines

Next, the expression of Smurf2 in HCC tissues, adjacent non-tumor liver tissues, and human HCC cell lines, was detected by western blot analysis. As shown in Figure 1e, a higher level of Smurf2 expression was found in adjacent non-tumor liver tissues, whereas HCC tissues and HCC cell lines.

#### 3.2.1 Smurf2 suppressed the migration, but not proliferation of human HCC cell lines

**Figure 2:**
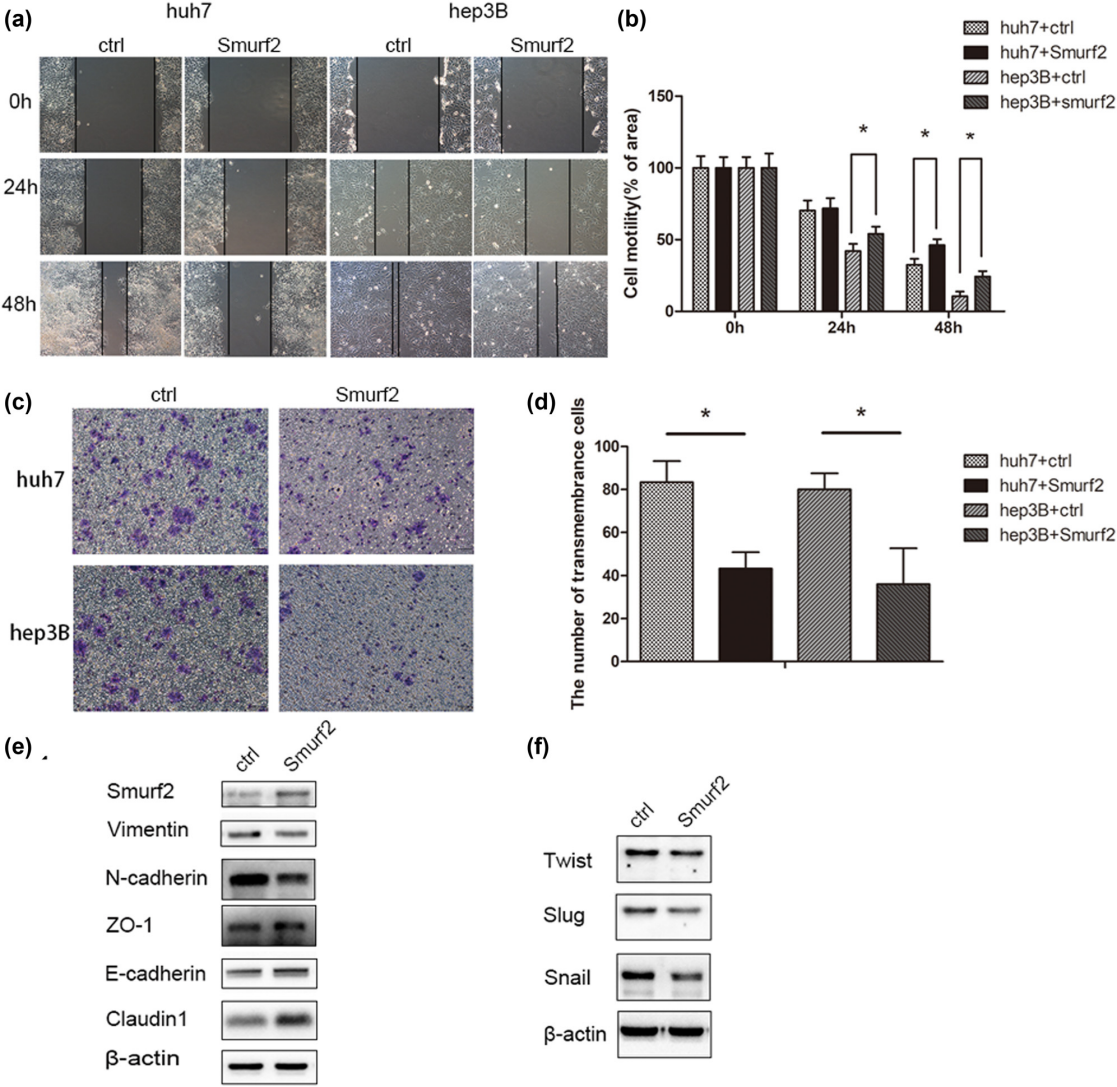
Smurf2 suppressed the migration of human HCC cell lines. (a and b) Wound healing assay in Hep3B-Smurf2 and Huh7-Smurf2 cells, *P < 0.05; (c and d) transwell migration assay of Hep3B-Smurf2 and Huh7-Smurf2 cells, *P < 0.05; (e) the immunoblotting of E-cadherin, ZO-1, Claudin-1, N-cadherin, and Vimentin protein levels in Huh7-Smurf2 cells; (f) the immunoblotting of EMT transcription factors protein levels in Huh7-Smurf2 cells.

To study the function of Smurf2 in HCC, Hep3B and Huh7 cells were stably transfected with Smurf2 using lentivirus methods, and wound-healing migration assays and transwell examinations were used to assess the effect of Smurf2 expression on HCC cell migration. As shown in Figure 2a and b, in the wound healing study, migrated cell count was significantly lower in Hep3B-Smurf2 and Huh7-Smurf2 cells compared with the control cells after 24 and 48 h. Similarly, the transwell assays of the effect of Smurf2 on HCC cells migration showed that the number of transmembrane cells was significantly lower in the Smurf2 overexpression groups than in the control groups (Figure 2c and d). Therefore, the overexpression of Smurf2 remarkably inhibited HCC cell migration.

**Figure 3:**
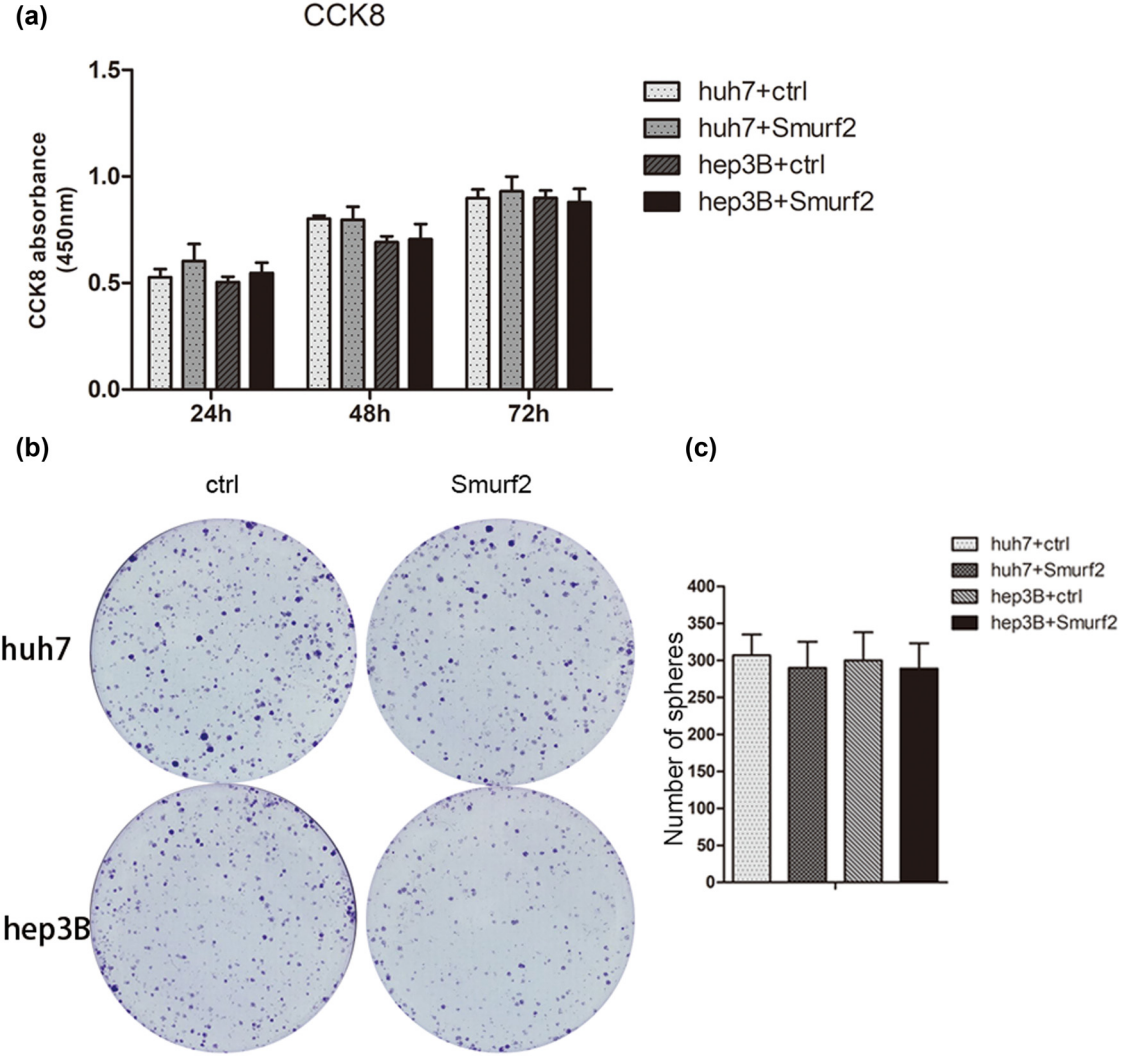
Smurf2 had no significant effect on the proliferation of HCC cell lines. (a) Detection of the proliferation of Hep3B-Smurf2 and Huh7-Smurf2 cells by CCK8 assay; *P < 0.05; (b and c) colony-forming assay of Hep3B-Smurf2 and Huh7-Smurf2 cells; *P < 0.05.

We also assessed cellular viability in Hep3B-Smurf2 and Huh7-Smurf2 cells. Two groups of HCC cells were incubated under normal conditions for 24–72 h, their viability was detected by CCK8 assay. There was no significant effect on cell proliferation compared with control cells (Figure 3a). After 2 weeks of culture under normal conditions, furthermore, no significant change was observed in the colony number of the Smurf2 group than the control group (Figure 3b and c).

Epithelial–mesenchymal transition (EMT) is one of the important characteristics of tumor metastasis. As important markers in the tumor EMT process, E-cadherin, ZO-1, Claudin-1 decreased, N-cadherin and Vimentin increased in expression. In addition, several transcription factors such as snail, twist, and slag are involved in the regulation of EMT and tumor metastasis. As indicated by immunoblotting performed to explore the expression of EMT markers in Smurf2-overexpression HCC cells. As showed in Figure 2e, western blotting data indicated that Smurf2 overexpression increased E-cadherin, ZO-1, Claudin-1 expression, and reduced N-cadherin and Vimentin expression in HCC cells. Additionally, Smurf2 overexpression significantly inhibited the expression of transcription factors such as twist, snail, and slug (Figure 2f). These data suggest that Smurf2 plays an inhibitory role in the EMT of HCC cells.

#### 3.2.2 Smurf2 interacts with and degrades Smad2 in HCC cell lines

**Figure 4:**
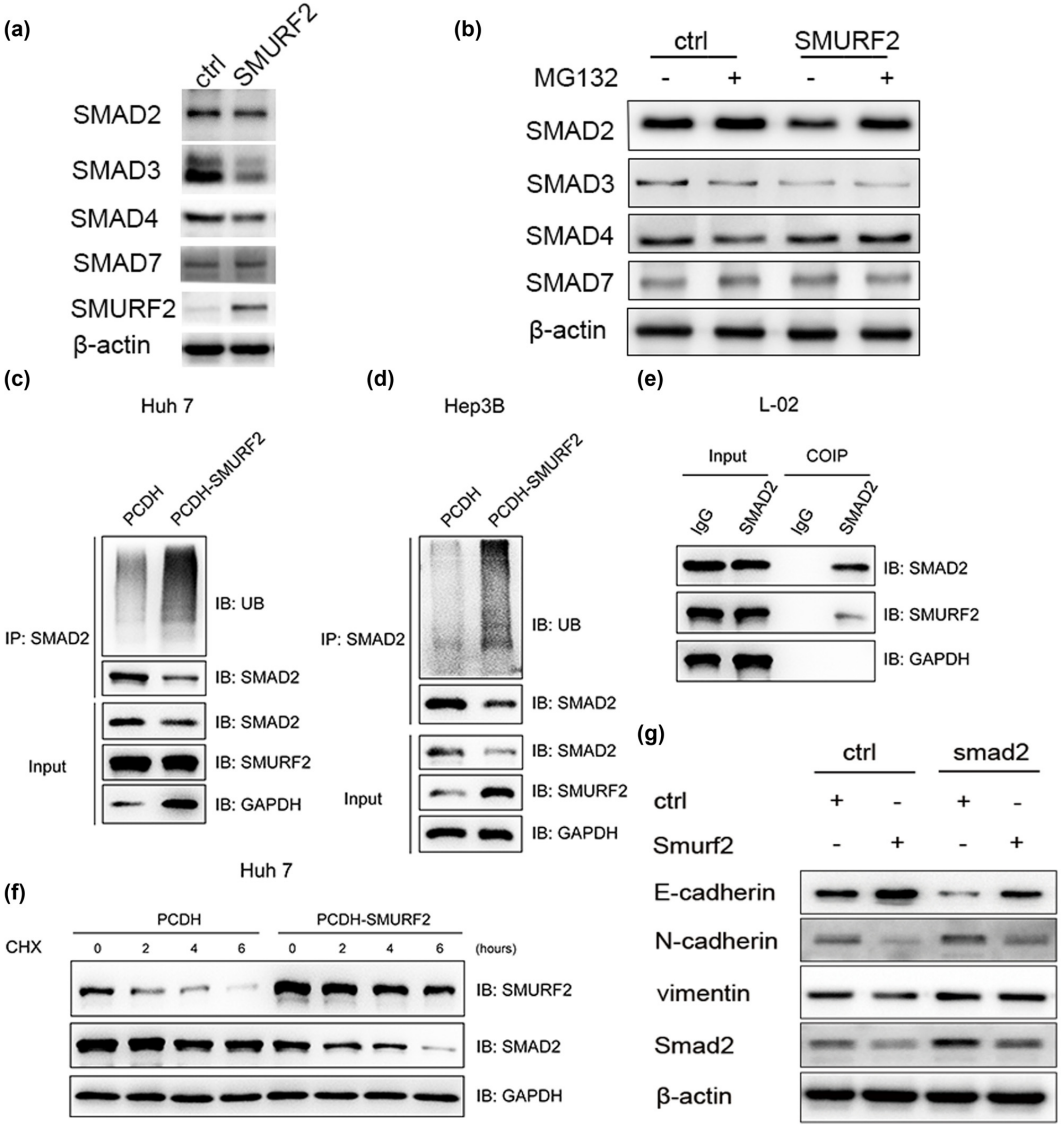
Smurf2 promoting the ubiquitination and degradation of Smad2. (a) The immunoblotting of Smurf2 and Smad2, Smad3, Smad4, and Smad7 protein levels in Huh7-Smurf2 cells. (b) Smurf2 promoting the degradation of Smad2 through proteasome; Huh7-Smurf2 cells treated with the proteasome inhibitor MG132 (2 µM) for 8 h; the protein levels of Smad2, Smad3, Smad4, and Smad7; (c and d) Smurf2 promoting the ubiquitination of Smad2; huh7 and hep3B cells treated with PTCDH-Smurf2, immunoprecipitated with anti-Smad2 antibody and subjected to immunoblotting analysis using indicated antibodies. (e) Co-IP assay to verify the interaction between Smurf2 and Smad2 in L02 cells. (f) Huh7 cells, with or without Smurf2 overexpression, were treated with protein synthesis inhibitor CHX (100 µg/mL) to block protein synthesis. immunoblotting was performed to detect Smad2 levels at different time points. (g) Smurf2 down-regulates the expression of Smad2 and inhibits EMT of HCC cells.

Next, we explored the mechanism of Smurf2 inhibiting the EMT process in HCC. TGF-β pathway plays an important role in EMT and metastasis of cancer cells [15]. Smurf2 is an E3 ubiquitin ligase, which plays a ubiquitination role by binding with the specific substrates, leading to ubiquitination degradation of the target proteins. It has been reported that Smurf2 is the specific E3 ligase involved in TGF-β signaling. Smurf2 affects TGF-β signaling pathways by regulating Smad proteins ubiquitination and degradation [10]. Next, we examined whether these Smad proteins are ubiquitination substrates of Smurf2 in HCC cells. As shown in Figure 4a, Smad2, Smad3, and Smad4 may be a substrate of Smurf2-mediated ubiquitination (Figure 4a). Next, we tested whether ubiquitin and proteasome-dependent pathways are involved in the interaction between Smurf2 and Smad2, Smad3, and Smad4 separately. It was observed that Smad2 was stabilized by proteasome inhibitor MG132 and was no longer affected by overexpression of Smurf2, which suggested that Smurf2 induced Smad2 degradation through ubiquitin–proteasome pathway rather than Smad3 or Smad4 (Figure 4b).

Previous studies have shown that Smurf2 could be combined with Smad2 affects the stability of Smad2 at the post-translational level. To determine whether Smurf2 mediated Smad2 decline is due to post-translational degradation, we treated the cells with proteasome inhibitor MG132. As shown in Figure 4b, the suppressive effects of Smurf2 on Smad2 protein were weakened after treatment with 2 µM MG132 for 8 h, suggesting that Smurf2 inhibits Smad2 by proteasome degradation. To investigate the mechanism of Smurf2 regulating Smad2 expression, first ubiquitination assay *in vitro* was performed. As expected, the ubiquitination of Smad2 was significantly increased in huh7 and hep3B cells treated with PTCDH-Smurf2 when compared with that of the PTCDH groups (Figure 4c and d). Next, we used a Co-IP assay to verify the interaction between Smurf2 and Smad2 in L02 cells. As shown in Figure 4e, Smurf2 and Smad2 are directly combined. Then, Huh7 cells, treated with PTCH-SMURF2 or PTCH, were treated with or without the use of protein synthesis inhibitor cyclohexanolamide (CHX, 100 µg/mL). Overexpression of Smurf2 significantly increased the degradation rate of Smad2 (Figure 4f). These results confirmed that Smurf2 could promote the ubiquitination and degradation of Smad2. We also examined whether Smurf2 inhibited EMT by Smad2. The inhibitory effect of EMT resulting from overexpression of Smurf2 was reversed by overexpression of Smad2 (Figure 4g). These data indicated that Smurf2 inhibited EMT of HCC via Smad2.

### 3.3 Smurf2 inhibits tumor metastasis *in vivo*

**Figure 5:**
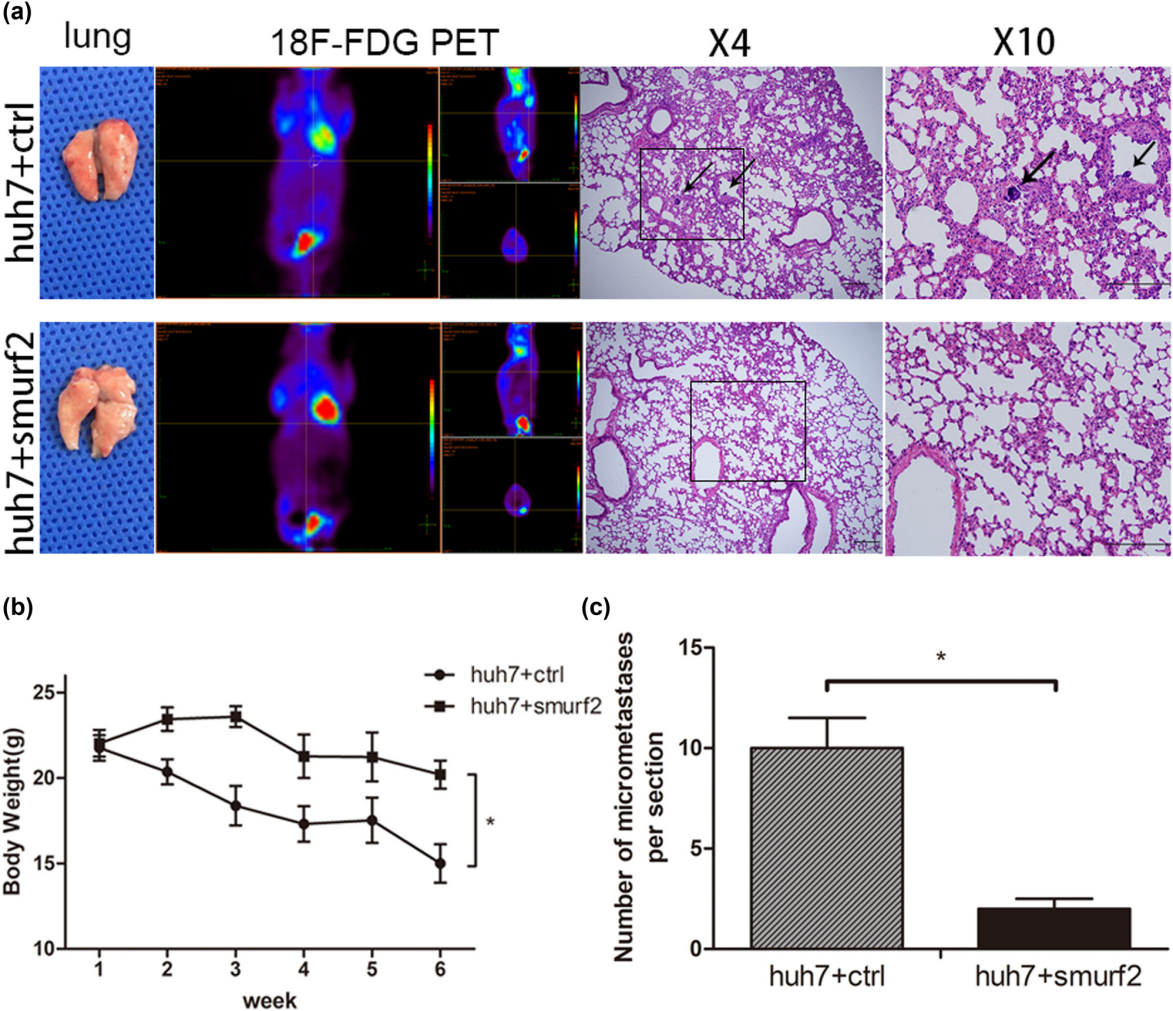
Smurf2 suppressing the HCC tumor metastasis *in vivo*. (a) Representative images of lungs and PET scan of different groups (white circles represent suspected lesions), and representative images of H&E staining of metastatic lung nodules from different groups. (b) Mouse weight was measured once a week at the indicated time points after injection with Huh7-Smurf2 or control cells. (c) Metastatic nodules in lungs of orthotopic xenograft mice model calculated; Values are presented as mean ± SD; *P < 0.05.

In addition, the effect of Smurf2 overexpression on HCC was further confirmed *in vivo*. When 12 mice were equally divided into two groups: Smurf2 overexpression and NC group, two groups of cells were injected into the nude mice through the tail vein (*n* = 6, per group). Six weeks later, all the mice were sacrificed. As shown in Figure 5b, compared with the control group, the weight of mice in the Smurf2 overexpression group increased significantly, while the weight of mice in the control group was significantly lower than that in the overexpression group. The result of PET/computed tomographic scan of lung and H&E staining of lung tissue showed that Smurf2 overexpression markedly reduced the number of lung metastases *in vivo* (Figure 5a and c), suggesting that Smurf2 could inhibit the metastasis of HCC *in vivo*.

## 4 Discussion

In this study, we found that Smurf2, a HECT-type E3 ubiquitin ligase, is a tumor suppressor of HCC. We studied the role of Smurf2 in regulating the EMT and migration of HCC through ubiquitin-dependent Smad2 degradation. Therefore, we found that the expression of Smurf2 in HCC was significantly lower than that in adjacent normal tissues. A low level of Smurf2 expression is significantly associated with unfavorable clinical features, such as tumor thrombus (Table 1, *P* = 0.045), and indicated a poor prognosis of HCC patients. Patients with higher Smurf2 expression had longer OS and DFS. Similarly, the expression of Smurf2 in HCC tissue and HCC cell lines was significantly lower than that in a normal liver tissue. Therefore, the low expression of Smurf2 in HCC may be a potential indicator of poor clinical prognosis. In addition, we also confirmed the inhibitory effect of Smurf2 on EMT and migration of HCC. We found that overexpression of Smurf2 markedly inhibited EMT and migration of HCC and significantly repressed lung metastasis of HCC *in vivo*. In addition, we verified the evidence that Smurf2 reduced Smad2 level and promoted Smad2 degradation by enhancing the ubiquitin-dependent proteasome pathway.

Smurf2 consists of an N-terminal C2 domain, three WW domains, and an evolutionarily conserved C-terminal HECT domain [5]. C2 domain mediates the binding of Smurf2 to the intracellular membrane. Smurf2 usually interacts with other proteins through the WW domain, mainly through the PPxY or LPxY motif of the substrate [16]. Smurf2 is thought to act as a tumor promoter or suppressor by regulating some proteins involved in tumorigenesis under different conditions [10]. The biological functions of Smurf2 and its related regulatory proteins are crucial for cancer progression and cancer treatment strategies.

It has been reported that Smurf2 plays a dual role in cancer as both tumor promoter and suppressor by regulating the protein stability in the process of tumorigenesis and development [17,18]. The expression of Smurf2 is closely related to the occurrence, development, and metastasis of the tumor. It is reported that the high expression of Smurf2 is closely related to bone metastasis of prostate cancer [19]. In pancreatic cancer, overexpression of Smurf2 inhibits TGF-β to mediate EMT [20]. Smurf2 knockout promotes the migration and bone metastasis of breast cancer [21]. This is similar to our results of Smurf2 inhibiting the migration and lung metastasis of HCC. In our research, Smurf2 mainly plays a tumor suppressor effect in HCC, which is different from the tumor-promoting effect in some other cancers [22,23]. This may be related to the different distribution of Smurf2 in cells. Mechanistically, the decrease in the nuclear pool of Smurf2 and increase in its cytoplasmic abundance could change the Smurf2’s access to its protein substrates, which include both tumor suppressor and oncogenes. The decrease in the nuclear pool of Smurf2 would diminish its ability to negatively regulate the pro-tumorigenic factors residing in the nucleus, while increased Smurf2 abundance in the cytoplasm would facilitate the cancer-promoting pathways, including EGFR-induced and KRAS-mediated signaling pathways and, suggestively, the WNT/β-CATENIN pathway [18]. Therefore, Smurf2 plays different tumor-promoting or anti-tumor effects in different cancers which require further study.

Many previous studies have reported that EMT has become the main factor of tumor malignancy, because EMT contributes to the motility and invasiveness of tumors, leading to distant metastasis [24]. The current results confirmed that the low expression of Smurf2 was closely related to the formation of tumor thrombus (*P* = 0.045). Overexpression of Smurf2 induced the inhibition of EMT in HCC. However, Smurf2 had no significant relationship with the proliferation and clonal ability of HCC.

EMT-inducing transcription factors play a major role in EMT, including snail, slug, and Twist1/2 [25]. EMT program organized by EMT-inducing transcription factors can endow cancer cells with several characteristics necessary for malignant progression, including tumor initiation, motility, diffusibility, and resistance chemotherapy [26,27]. For example, snail and ZEB2 activate the expression of matrix metalloproteinases, promote the degradation of the basement membrane, and promote cell invasion. In the present study, we examined the relationship between Smurf2 and EMT inducible transcription factors. Smurf2 overexpression inhibited the expression of snail, slug, and Twist1/2, which indicated that Smurf2 affected EMT of HCC and inhibited tumor progression by affecting EMT inducible transcription factors.

TGF-β pathway plays a central role in inducing EMT in different tissue types [28]. TGF-βs bind to complexes of TGF-β receptor type 1 (TGFβR1) and TGFβR2, leading to the phosphorylation of Smad2 and Smad3, which proceed to form complexes with Smad4. Smad mediates TGF-β-induced EMT by inducing the expression of E-cadherin transcriptional repressors, such as snail1, slug, ZEB1/2, or twist [29]. Previous studies identified Smurf2 ubiquitin substrates, including Smad1, Smad2/3, Smad5, Smad6/7, and TGF-βR1 [30]. Smad2 can interact through the PPxY motif with the WW domains of Smurf2 [31]. Smad7 may activate Smurf2 by enhancing the interaction of Smurf2 [32]. In the present study, we have found that Smurf2 overexpression inhibits EMT-induced transcription factors snail, slug, and Twist1/2 expression, thereby inhibiting EMT of HCC. Therefore, whether Smurf2 affects TGF-induced EMT by affecting Smad activity remains unknown. We detected the expression of Smad2, Smad3, Smad4, and Smad7 after overexpression of Smurf2 in HCC. And then, we detected the expression of Smad2, Smad3, Smad4, and Smad7 after using proteasome inhibitors MG132. We found that Smurf2 had the most significant effect on Smad2 in HCC. Smurf2 promoted the degradation of Smad2 through the enhancement of ubiquitination. However, the signal pathways in HCC are very complex. Whether Smurf2 can inhibit EMT by influencing other pathways requires further study.

In summary, we assumed that Smurf2 could be one of the important markers for HCC. Our findings elucidated that the downregulated expression of Smurf2 in HCC conferred poor clinical outcomes. We provided evidence to support that Smurf2 inhibits HCC EMT by promoting the degradation of Smad2 through the enhancement of ubiquitination. These data may provide a new approach for the treatment of HCC.

## 5 Conclusion

Our research shows that Smurf2 could inhibit EMT of HCC by increasing the degradation and ubiquitination of Smad2, which reveals a new mechanism of Smurf2 in the development of HCC and provides an effective target for the treatment of HCC.

## Supplementary Material

Supporting Information 1

Supporting Information 2
