# Supplementary material for: Smurf2 suppresses the metastasis of hepatocellular carcinoma via ubiquitin degradation of Smad2
Source: Open Med (Wars). 2022 Feb 24;17(1):384–96. doi: 10.1515/med-2022-0437 (PMC8874264; doi:10.1515/med-2022-0437)
Supplement: Supporting Information 2 [file med-2022-0437-appendix-s2.pdf]

```
GET
FILE= 'C :\Users\song\Desktop\smurf2 response2\smurf2.sav'.
DATASET NAME 1 WINDOW= FRONT.
T-TEST PAIRS= tumor WITH paratumor (PAIRED)
/CRITERIA= CI(.9500)
/MISSING= ANALYSIS.
```

T-TEST PAIRS

[1] C:\Users\song\Desktop\smurf2 response2\smurf2.sav

Paired Samples Statistics

		Mean	N	Std.Deviation	Std.Error Mean
pair 1	tumor	4.6864	220	3.34839	.22575
	paratumor	6.8682	220	3.04995	.20563

Paired Samples Correlations

		N	Correlation	Sig.
Pair 1	tumor & paratumor	220	.981	.000

Paired Samples Test

		Paired Differences			t	df	Sig. (2-tailed)
		Mean	Std.Deviation	Std.Error Mean			
Pair 1	tumor - paratumor	-2.18182	.69810	.04707	-46.357	219	.000
